# A novel meropenem dosing strategy for Outpatient Parenteral Antimicrobial Therapy (OPAT): a pilot pharmacokinetic/pharmacodynamic study in non-critically ill patients

**DOI:** 10.1128/aac.00372-26

**Published:** 2026-06-12

**Authors:** Caroline Briquet, Perrin Ngougni Pokem, Xin Liu, Gert-Jan Wijnant, Olivier Cornu, Halil Yildiz, Alexia Verroken, Jason A. Roberts, Jean Cyr Yombi, Françoise Van Bambeke

**Affiliations:** 1 Cliniques Universitaires Saint-Luc, Antimicrobial Stewardship 70492, Brussels, Belgium; 2 Department of Pharmacy, Cliniques Universitaires Saint-Luc 70492, Brussels, Belgium; 3 UCLouvain, Louvain Drug Research Institute, Pharmacologie Cellulaire et Moléculaire 508610 , Brussels, Belgium; 4 Clinical Microbiology Department, Cliniques Universitaires Saint-Luc 70492, Brussels, Belgium; 5 Faculty of Health Medicine and Behavoural Science, The University of Queensland, University of Queensland Centre for Clinical Research 1974, Brisbane, Australia; 6 Laboratory of Clinical Microbiology, KU Leuven 26657, Leuven, Belgium; 7 Department of Orthopaedic and Trauma Surgery, Cliniques Universitaires Saint-Luc, UCLouvain 70492, Brussels, Belgium; 8 Department of Internal Medicine and Infectious Diseases, Cliniques Universitaires Saint-Luc, UCLouvain 70492, Brussels, Belgium; 9 Herston Infectious Diseases Institute (HeIDI), Metro North Health, Brisbane, Australia; 10 Department of Pharmacy, Royal Brisbane and Women’s Hospital, Brisbane, Australia; 11 Department of Intensive Care Medicine, Royal Brisbane and Women’s Hospital, Brisbane, Australia; 12 Division of Anesthesia Critical Care and Emergency and Pain Medicine, UR UM 103, University of Montpellier, Nimes University Hospital, Nimes, France; Providence Portland Medical Center, Portland, Oregon, USA

**Keywords:** meropenem, OPAT, PK/PD, population pharmacokinetics, Monte Carlo simulations

## Abstract

Meropenem is a valuable option for outpatient parenteral antimicrobial therapy (OPAT) due to its broad-spectrum activity, including multidrug-resistant Gram-negative bacteria, particularly when no oral alternatives exist. However, its conventional thrice-daily dosing poses logistical challenges for home administration, as the third dose typically falls during nighttime. This prospective pharmacokinetic study evaluated the adequacy of a novel daytime-only meropenem regimen adapted for OPAT in non-critically ill hospitalized patients. Twenty-two patients receiving meropenem 2 g every 8 h were switched, once clinically stable, to a thrice-daily, 6-hourly regimen compatible with daytime administration only. Meropenem was infused over 20 min. At steady state, unbound meropenem concentrations were measured for both regimens. These data informed a population pharmacokinetic model and Monte Carlo simulations to compare the probability of achieving predefined PK/PD targets. Both the conventional and OPAT-adapted regimens achieved a probability of target attainment (PTA) >90% for 40% *f*T >MIC at MIC ≤4 mg/L, exceeding the EUCAST/IDSA susceptibility breakpoint of 2 mg/L. Extending the infusion duration to 3 or 5 h with the OPAT-adapted schedule restored PTA >90% for MIC ≤8 mg/L. However, trough concentrations after the evening dose were lower with the OPAT-adapted regimen than with the standard regimen (1–2 vs 3–5 mg/L, depending on infusion duration). This may increase the risk of resistance selection despite the post-antibiotic effect of meropenem. With this caveat, OPAT regimens, with infusion duration adapted to MIC, may enable practical daytime-only meropenem administration, avoiding the need for overnight nursing visits.

## INTRODUCTION

Outpatient parenteral antimicrobial therapy (OPAT) has expanded in recent years, offering a cost-effective alternative to inpatient care when oral therapies are not available. OPAT reduces the risk of healthcare-associated infection and enhances patients’ quality of life (1). A wide range of parenteral antimicrobial agents can be used in OPAT, depending on the susceptibility profile of the causative pathogen and the site of infection. Among these, carbapenems can be a valuable choice for infections caused by multi-drug-resistant Gram-negative bacteria. However, their use presents specific challenges (2), especially in the case of meropenem, which is the only carbapenem commercially available in Belgium.

Like other β-lactam antibiotics, meropenem exhibits time-dependent bactericidal activity. Its therapeutic efficacy is primarily determined by the proportion of the dosing interval during which the unbound (free) plasma concentration remains above the minimum inhibitory concentration (MIC) of the target pathogen (*f*%T > MIC), with possible contributions from post-antibiotic effects (PAEs) (3). Notably, carbapenems are unique among β-lactams in that they exhibit prolonged PAE (approximately 2–4 h) against Enterobacterales and *Pseudomonas aeruginosa* (4, 5). This feature is associated with lower *f*%T > MIC thresholds compared to other β-lactams. According to the EUCAST (European Committee on Antimicrobial Susceptibility Testing) rationale document for meropenem (6) based on pharmacodynamic studies in a variety of models of infection by Enterobacterales and *P. aeruginosa,* bacteriostasis is achieved when the free drug concentrations exceed the MIC (*f*%T > MIC) for 25–40% of the dosing interval, while a 1-log_10_ bacterial killing requires *f*%T > MIC values between 35% and 55%.

From a pharmacokinetic/pharmacodynamic (PK/PD) perspective, continuous infusion is considered the optimal mode of administration for β-lactams, as it maximizes the time the drug concentration remains above the MIC and may improve clinical outcomes (7, 8). However, meropenem lacks sufficient stability to permit extended administration over 12 or 24 h: its chemical stability in solution does not reliably exceed 5–8 h at room temperature or 33°C for concentrations ranging from 15 to 25 mg/L, and can even fall down to 1.5 h in more concentrated solutions (64 mg/mL) at 37°C (9–12). This stability also varies depending on the specific generic formulation used (10). This limitation is particularly relevant when the drug is administered through portable infusion (elastomeric or electronic pumps), commonly used in OPAT, potentially exposed to temperatures >25°C.

A thrice-daily (TID) schedule is therefore adopted, but presents logistical challenges for outpatient administration, particularly regarding the third dose, which typically falls around midnight. One recent study reports the safety and efficacy of meropenem given as continuous infusion with renewal of the bag every 8 h in a pediatric population (13). This supposes that patients or their parents were trained to handle antibiotic self-administration and catheter flushing. However, the patient population selected for the OPAT program is not always suited to self-managing pump replacements (2, 14). These patients typically require short-term, non-repetitive antibiotic therapy; they are also often older, present with acute infections, and have multiple comorbidities. As a result, they usually depend on home nurse visits. In the hospital setting, meropenem is administered TID at 8-h intervals (*quaque* 8 h or q8-8-8 h), as short infusion over 20 min or prolonged infusion lasting up to 3 h in patients with normal renal function. Such a regimen is practically challenging in an OPAT scenario.

**Fig 1:**
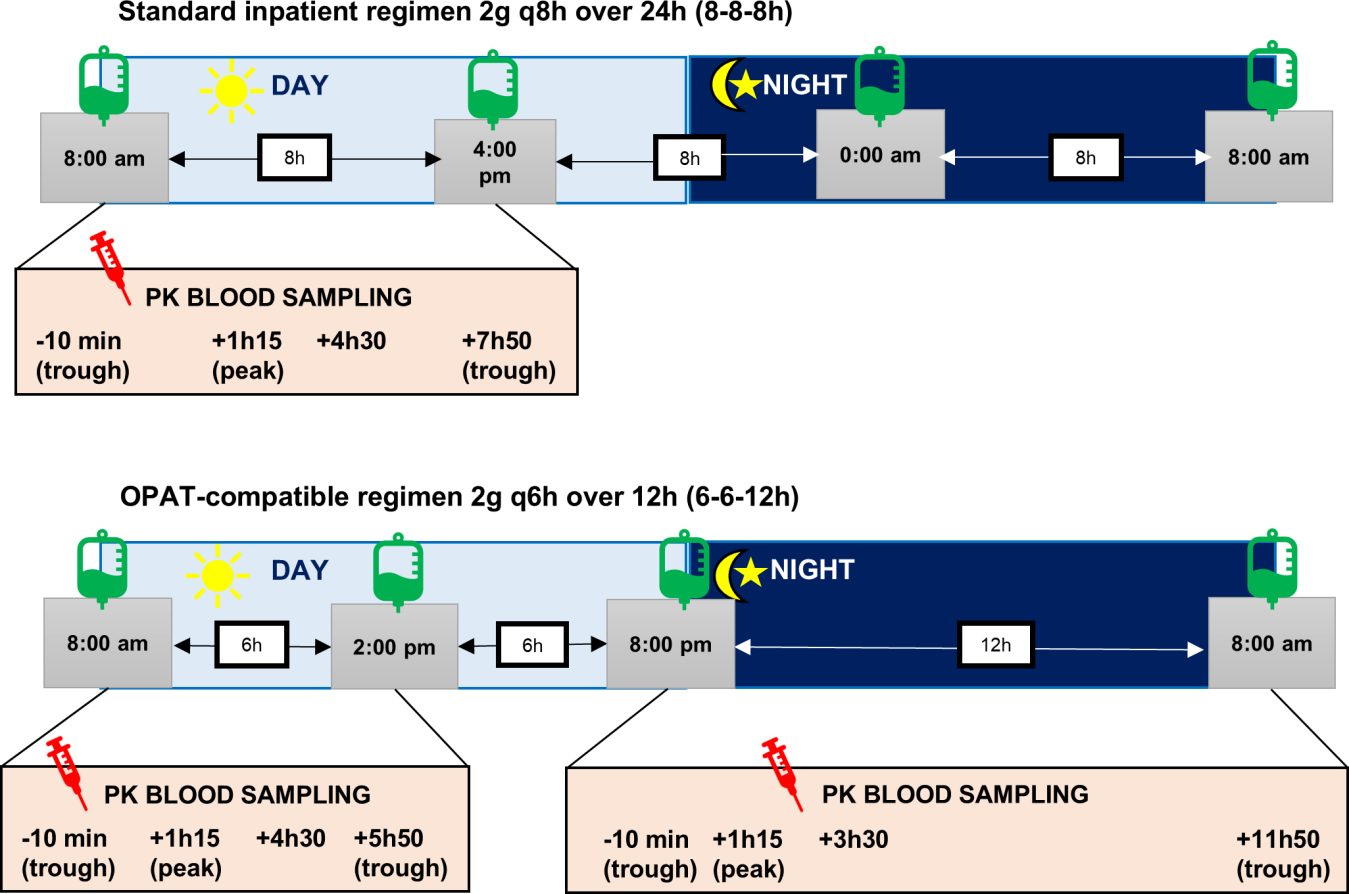
Graphical representation of the scheme of administration of meropenem (represented by the infusion bag), according to the conventional inpatient regimen (TID, q8-8-8 h; top) or to the OPAT-compatible regimen (TID, q6-6-12 h; bottom). The box below each timeline indicates the timing of blood sampling (signified by the syringe) for pharmacokinetic studies. Four samples were collected over each dosing interval.

This prospective pharmacokinetic study in hospitalized non-critically ill patients aims to evaluate the dosing adequacy of a novel daytime-only meropenem dosing regimen for use in an OPAT setting. In the proposed regimen, the same total daily dose of 6 g (as recommended for difficult-to-treat infections [15]) is administered as three 2 g doses over a 12-h period (*quaque* 6 h over a 12-h period, i.e., 6-6-12 h), with the evening dose scheduled earlier to avoid overnight nursing visits (Fig. 1) .

We compared the traditional inpatient regimen (q8-8-8 h) with the proposed alternative, OPAT-adapted schedule (q6-6-12 h), using population pharmacokinetic/pharmacodynamic model analysis (POP-PK/PD) and Monte Carlo simulations in order to assess the feasibility of this modified dosing strategy in achieving appropriate pharmacodynamic targets for meropenem in the OPAT setting.

## RESULTS

### Study population and sampling

Twenty-two non-critically ill patients were enrolled in the study and contributed a total of 246 blood samples (from all patients for the conventional scheme and 20 patients for the OPAT-adapted scheme [no samples for two patients due to the necessity to remove their central line catheter]). Four concentrations were excluded from the analysis: one with uncertain sampling time and three, identified as outliers using the conservative Tukey 3.5 × IQR rule, a distribution-independent method that robustly identifies values unlikely to arise from the main body of the data without assuming normality. Specifically, observations lying below Q1−3.5 × IQR or above Q3 + 3.5 × IQR, where IQR is the interquartile range, were considered extreme. Patient characteristics are summarized in Table 1. No patient had an eGFR lower than 60 mL/min.

**TABLE 1:** Characteristics of patients and infections^*d*^

Patients’ characteristics		Median (range)
Male/Female		*N* = 12/10
Age (years)		61.5 (26–82)
Weight (kg)		85 (44–155)
BMI^*a*^ (kg/m^2^)		26.35 (16.8–47.9)
eGFR^*b*^		102 (64–234) Mean: 108.1
Patients with no renal insufficiency (GFR > 90 mL/min/1.73 m^2^), *N* = 16		113 (91–234)
Patients with mild renal insufficiency (GFR = 90–60 mL/min/1.73 m^2^), *N* = 6		78 (64–87)
Infection type	Bone	*N* = 15 (68%)
	Intra-abdominal - Liver - Pancreas - Colorectal	*N* = 5 (23%) *N* = 3 *N* = 1 *N* = 1
	Skin and soft tissue	*N* = 2 (9%)
CRP^*c*^ (mg/L) per infection type	Bone	73.2 (9.1–304.9)
	Intra-abdominal	147.5 (60.7–212.5)
	Skin and soft tissue	173.05 (168.5–177.6)

^*a*^ BMI, body mass index.

^*b*^ eGFR, estimated glomerular filtration rate using CDK-EPI equation.

^*c*^ CRP, C-reactive protein; baseline values measured at the onset of the infection or at the start of meropenem therapy.

^*d*^ Data are expressed in median [range], unless specified otherwise.

### Microbiological data

Twenty-six strains were isolated, with the most common bacteria being *Escherichia coli* (23.7%), *Klebsiella pneumoniae* (19.23%), *Enterobacter cloacae* complex (15.4%), *P. aeruginosa* (7.7%), and *Serratia marcescens* (7.7%). The meropenem MIC was ≤0.125 mg/L for 18 isolates, 2 mg/L for 5 isolates, and 0.25, 0.5, and 1 mg/L, respectively, for the 3 last isolates (Table S1).

### Population pharmacokinetic modeling

The concentration-time profiles were best described by a two-compartment model with first-order elimination. Inter-individual variation (IIV) was estimated for clearance and volume of distribution of the central compartment. A combined (additive plus proportional) residual error model provided the best fit for unbound plasma concentrations. No covariate met the criteria for inclusion during covariate analysis. Population pharmacokinetic parameter estimates, along with results from 1,000 bootstrap replicates, are presented in Table 2. GOF plots of the final model and VPC plots are presented in Fig. S1 and S2.

**TABLE 2:** Estimates of the population pharmacokinetic parameters

Parameter^*a*^	Estimate (%RSE) [shrinkage %]	Bootstrap median (95% CI)
Fixed effects		
CL (L/h)	11.7 (11.1)	10.6 (5.72–14.5)
V_1_ (L)	23.6 (11.9)	20.6 (2.58–30.1)
Q (L/h)	1.98 (39.5)	2.45 (0.61–7.90)
V_2_ (L)	5.44 (17.3)	6.21 (3.41–72.5)
Random effects		
IIV_CL (%)	51.7 (17.1) [−1.08]	49.7 (16.1–71.1)
IIV_V_1_ (%)	42.9 (25.2) [12.1]	50.3 (23.2–136)
Corr_V_1__Cl	0.76 (18.0)	0.72 (0.53–0.9)
Residual variability		
Additive (mg/L)	0.16 (48.5)	0.17 (0.017–0.59)
Proportional	0.41 (8.26)	0.40 (0.29–0.54)

^*a*^ CI, confidence interval; CL, plasma clearance; Corr_V_1__CL, correlation between IIV of clearance and volume of distribution in the central compartment; IIV, inter-individual variability; Q, intercompartmental clearance between compartment 1 and compartment 2; RSE, relative standard error; V_1_, volume of distribution in the central compartment; V_2_, volume of distribution in the peripheral compartment.

**Fig 2:**
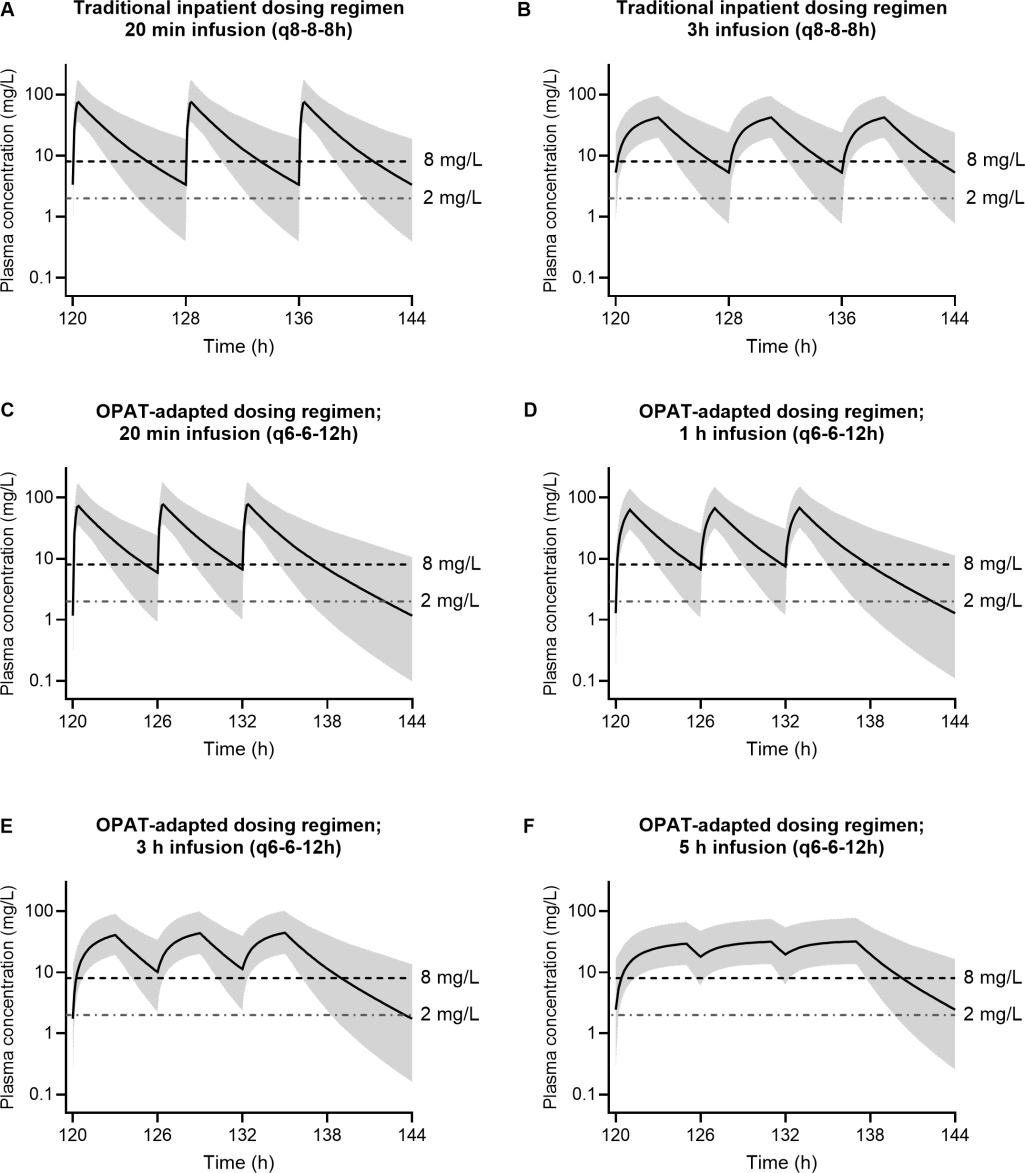
Simulated unbound plasma concentration-time profiles of meropenem given at 6 g/day using different dosing regimens: traditional inpatient dosing (TID, q8-8-8 h) with 20 min (**A**) or 3 h (**B**) infusion, or OPAT adapted regimens (TID, q6-6-12 h) with 20 min (**C**), 1 h (**D**), 3 h (**E**), or 5 h (**F**) infusions. The solid line represents the median of 1,000 simulations, and the gray band, the 95% confidence interval. The dashed lines represent MIC of 2 and 8 mg/L (IDSA and EUCAST susceptibility breakpoints [increased dose for EUCAST]).

Monte Carlo simulations based on the final pop-PK model were conducted to assess unbound plasma concentration-time profiles and PK/PD target attainment (PTA) across six dosing regimens and varying MIC scenarios. For the standard inpatient regimen (Fig. 2A; Table S2), unbound concentrations remained above MIC values of 2 and 8 mg/L during 24 h [95% CI: 14 to >24] and 16 h [14–24], respectively. When the infusion was extended to 3 h (Fig. 2B; Table S2), concentrations remained above the same thresholds for 24 h [13 to >24] and 20 h [12 to >24], respectively.

Under the corresponding OPAT-adapted regimen with a 20-min infusion (Fig. 2C; Table S2), unbound concentrations remained above MIC of 2 and 8 mg/L for 22 h [14 to >24] and 16 h [9 to >23]. Prolonging the infusion to 1 h (Fig. 2D) yielded similar results (22.5 h [16 to >23] and 16 h [9 to >24]), whereas a 3-h infusion (Fig. 2E) increased the time above MIC to 23.5 h [18 to >24] for 2 mg/L and 19 h [12 to >24] for 8 mg/L. Further prolongation to a 5-h infusion, still compatible with meropenem stability and feasible between two daytime nurse visits, maintained unbound concentrations above 2 mg/L during 24 h [20 to > 24] and above 8 mg/L during 20 h [17 to >24] (Fig. 2F).

The trough concentration at 8 h post-dose with the conventional regimen was 3.2 mg/L [0.4–19] and 5.3 mg/L [0.8–24] for infusion times of 20 min and 3 h, respectively. With the OPAT-adapted regimen, the concentration 12 h after the evening dose decreased to 1.2 mg/L [0.01–13], 1.3 mg/L [0.01–13], 1.7 mg/L [0.04–15], or 2.5 mg/L [0.3–16.5] depending on the infusion duration (Table S2).

**Fig 3:**
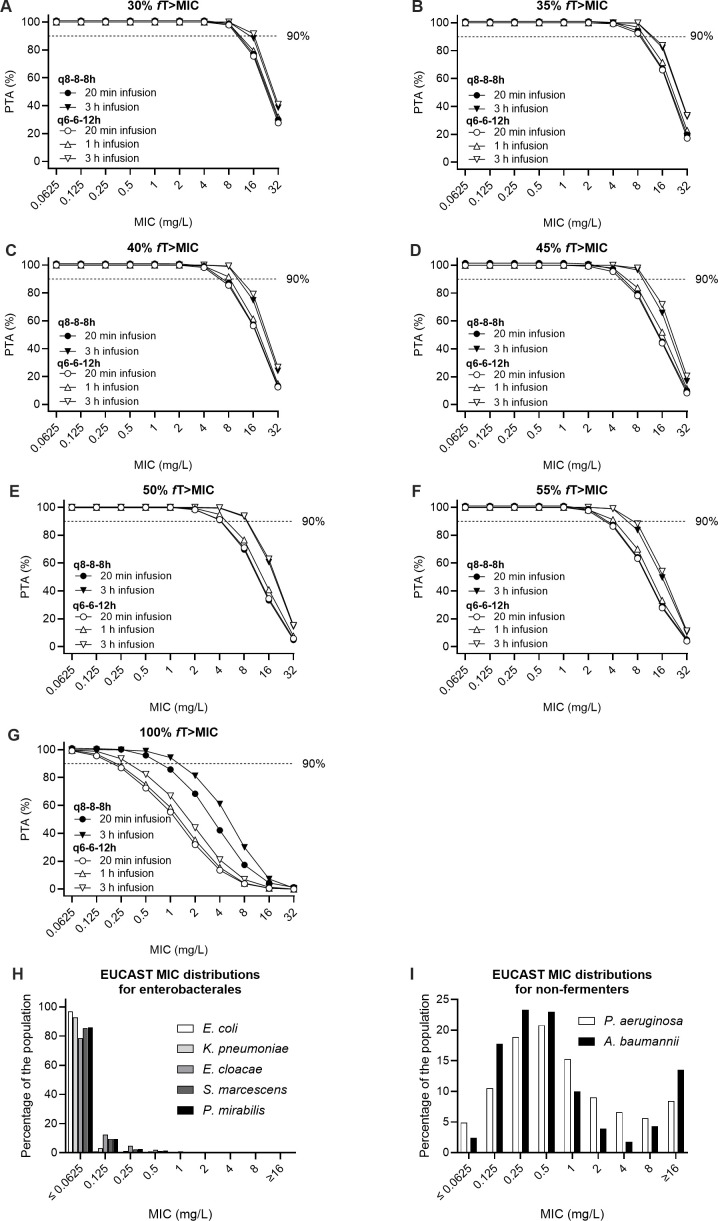
Monte Carlo simulations and probability of target attainment (PTA) analysis obtained from 1,000 simulations for meropenem given at 6 g/day using different dosing regimens: traditional inpatient dosing (TID, q8-8-8 h) with 20 min (closed circles) or 3 h infusion (closed inverted triangles), or OPAT-adapted regimens (TID, q6-6-12 h) with 20 min (open circles), 1 h (open triangles), or 3 h (inverted triangles) infusions. Targets are defined as 30% *f*T > MIC (**A**), 35% *f*T > MIC (**B**), 40% *f*T > MIC (**C**), 45% *f*T > MIC (**D**), 50% *f*T > MIC (**E**), 55% *f*T > MIC (**F**), and 100% *f*T > MIC (**G**). Dashed lines represent a PTA of 90%. The MIC distributions from the EUCAST database for the most frequent Enterobacterales species and non-fermenters among species collected from the included patients are shown in panels **H** and **I,** respectively. Note that all patients in this study were infected by bacteria with MIC ≤ 2 mg/L according to inclusion criteria.

PTA was calculated for targets of 30%, 35%, 40%, 45%, 50%, 55%, and 100% *f*%T > MIC across MIC values up to 16 mg/L (the highest target being rather recommended for critically ill patients (16)). Simulations compared the traditional inpatient regimen (with 20 min and 3 h infusions) with OPAT-adapted regimens (with 20 min, 1 h, 3 h, and 5 h infusions). Results are summarized in Fig. 3 and Table S3. PTA exceeded 90% for PK/PD targets up to *f*%T > 50% and an MIC of 4 mg/L across all dosing regimens. At an MIC of 8 mg/L, a 90% PTA was achieved for various PK/PD targets, depending on the administration scheme: 35% *f*%T > MIC with a 20-min infusion (for both the conventional and OPAT-adapted dosing regimens), 40% *f*%T > MIC with a 1-h infusion (OPAT-regimen), 50% *f*%T > MIC with a 3-h infusion (for both the conventional and OPAT-adapted dosing regimens), and 55% *f*%T > MIC with a 5-h infusion (OPAT regimen). Of note, none of the dosing regimens achieved a 90% PTA for the target 100% *f*%T > MIC at MIC ≥ 2 mg/L corresponding to the susceptibility breakpoint. However, this target was reached for MIC up to 0.25 mg/L (OPAT-adapted dosing regimens and infusion durations ≤3 h), and up to 0.5 mg/L (conventional dosing regimen with short infusion or OPAT-adapted regimen administered over 5 h), or up to 1 mg/L (conventional dosing regimen with prolonged infusion). These MIC thresholds encompass most Enterobacterales (Fig. 3H), and 35% (3-h infusion) to 55% (5-h infusion) of non-fermenters (Fig. 3I).

## DISCUSSION

This study is, to the best of our knowledge, the first to examine, from a PK/PD perspective, the appropriateness of adapting the TID dosing regimen of meropenem over a 12-h interval in order to facilitate nursing for OPAT applications, while taking into account the limited stability of meropenem and its short half-life.

The present pop-PK model for the traditional dosing regimen aligns with previously published studies, thereby supporting the validity of the applied modeling approach. Although most (but not all) of the referenced studies concern critically ill patients (see, e.g., references 17–21), this study confirms that meropenem pharmacokinetics are best described by a two-compartment model. In contrast to these studies, renal function was not identified as a significant covariable, as (i) the included patient population did not exhibit renal impairment and (ii) renal function was limitedly spread in the global study population, in line with inclusion criteria. Similarly, although albumin was reported as a relevant covariate in previous work, albumin levels were not measured, but rather modeled unbound drug concentrations, rendering albumin levels irrelevant in this analysis. It is also worth mentioning that meropenem exhibits minimal protein binding (approximately 2%). Of note, Monte Carlo simulations based on the population model indicate that the conventional dosing regimen with a dose of 2 g q8 h is sufficient to achieve the pharmacodynamic target of 40% *f*%T > MIC for pathogens with MIC up to 4 mg/L or up to 8 mg/L with prolonged infusion over 3 h, which is consistent with most published studies (18, 20, 21). Moreover, simulations for lower PK/PD targets of 30–35% *f*%T > MIC (aimed at achieving a bacteriostatic effect) are consistent with the EUCAST breakpoint (8 mg/L), which specifically defines as susceptible, increased exposure a microorganism for which there is a high likelihood of therapeutic success when administering a high dose (as used in this study) (6).

Importantly, modifying the administration schedule from TID q8-8-8 h to q6-6-12 h does not impact PTA for these specific targets at MIC of 2 or 4 mg/L. This PTA-based assessment, however, does not address a distinct consideration related to the risk of resistance selection, which is better predicted by the Cmin/MIC ratio (22). The trough concentration after the evening dose was lower with the OPAT-adapted regimen (1.2 mg/L) compared to the conventional regimen (3.2 mg/L). This reduced exposure may thus favor resistance emergence for organisms with MIC ≥ 0.25 mg/L (vs 0.8 mg/L for the conventional regimen), given that maintaining trough levels at least fourfold above the MIC is considered as protective (22).

For time-dependent antibiotics that are unsuitable for continuous infusion due to their limited chemical stability like meropenem, prolonged infusion offers a viable strategy to enhance drug exposure (23–26). Accordingly, meropenem is also administered as a prolonged infusion over 3 h (27). Monte Carlo simulations indicate that applying this mode of administration to the OPAT-adapted regimen yields a higher PTA than the conventional inpatient schedule with 20-min infusions, and a PTA comparable to that achieved with the conventional 3-h infusion for targets up to 55% *f*T > MIC. Whether extending the infusion duration also helps to mitigate the risk of resistance selection for the OPAT-adapted regimen remains to be established: when meropenem is infused over 3 h or longer, Cmin values are ≥10 mg/L after the two diurnal doses but only reach 1.7–2 mg/L after the evening dose, for 3–5 h infusions, respectively. This intermittent drop in concentrations during the overnight interval may theoretically place drug exposure within the mutant selection window, a pharmacodynamic concept not captured by conventional *f*T > MIC targets. The clinical significance of these variations therefore warrants careful evaluation in clinical practice for key target pathogens, which may harbor different resistance mechanisms or propensity to develop resistance.

The optimal value of the PK/PD target remains a matter of ongoing debate, as it is strongly influenced by factors such as the severity of the infection (3), the patient’s immune status (28), or the site of the infection (4). It may also vary across β-lactam subclasses: carbapenems exhibit a post-antibiotic effect not seen with other β-lactams and therefore may not require as prolonged a duration of concentrations above the MIC to achieve optimal efficacy (29, 30). For these reasons, a broad range of PK/PD targets was considered, guided by the EUCAST rationale documents (6). In contrast, most studies conducted in non-critically ill patients generally focus on a target of 40% *f*%T > MIC. For this specific target, and considering that the vast majority of isolates of Enterobacterales and non-fermenters still exhibit MIC values ≤2 mg/L (see EUCAST MIC distributions in Fig. 3I), the data suggest that the OPAT-adapted regimen with 20-min infusion would be effective in most of the cases.

Yet, higher targets (typically ranging from 50% to 100% *f*T >1× MIC to 4× MIC) are desirable in deep-tissue or difficult-to-treat infections or in infections caused by microorganisms with MICs ≥2 mg/L, as well as in patients requiring prolonged antibiotherapy, in order to prevent emergence of resistance (3, 16, 31). Simulations suggest that none of the dosing regimens tested, including the conventional q8-8-8 h dosing administered with extended infusion, allow reaching a target at 100% *f*T > MIC in 90% of the population at the susceptibility breakpoint of 2 mg/L. These results challenge the suitability of meropenem in this clinical scenario more than the appropriateness of any particular administration schedule.

This study has some limitations. First, the study population did not include patients with renal insufficiency nor critically ill patients. This exclusion was intentional, based on two assumptions: (i) patients with high renal function are of particular interest for our research question because they are at greater risk of underdosing than those with renal insufficiency who experience higher β-lactam exposure and typically require only two doses per day, a simple regimen for nurses; (ii) critically ill patients are not eligible for home therapy. Second, while meropenem pharmacokinetic profiles and the probability of achieving specific PK/PD targets were characterized, this study was not designed or powered to evaluate clinical outcomes such as efficacy, safety, or the risk of resistance selection associated with the proposed dosing regimens.

To conclude, and with these caveats in mind, this work suggests that the studied alternative regimen (q6-6-12 h) is pharmacodynamically equivalent to the conventional regimen (q8-8-8 h) for pathogens with MIC ≤4 mg/L when using a 20-min infusion (if targeting *f*T > MIC up to 50%). Extending the infusion duration to 3 or 5 h allows coverage of pathogens with MIC of 1 or 2 mg/L if targeting 55% *f*T >4 xMIC, as required for difficult-to-treat infections in deep tissues. These two same dosing regimens cover pathogens with MIC up to 0.25 or 0.5 mg/L, respectively, when aiming for 100% *f*T > MIC. Based on EUCAST MIC distributions, this corresponds to coverage of 97% of enterobacterales and 35% of non-fermenters. Finally, to reach the stringent 100% *f*T > 4×MIC target that fully prevents resistance selection (22), 5-h extended infusions are needed to cover MIC ≤ 0.125 mg/L, that is, still 95% of Enterobacterales, but only 16% of non-fermenters.

Based on these results, dosing recommendations are presented in Table 3, and may serve as a foundation for further clinical validation aiming at studying efficacy, safety, emergence of resistance, or pharmacoeconomic impact of this approach. The proposed regimens may facilitate home-based administration by accommodating nursing constraints, thereby supporting broader implementation of OPAT with meropenem. Its use should, however, remain limited to hemodynamically and clinically stable patients, consistent with general OPAT recommendations (32), and to infections caused by microorganisms with MIC values up to 2 mg/L. More stringent thresholds (i.e., MIC up to 1 mg/L) should be applied for patients with deep-seated infections, for whom safer PK/PD targets are *f*T >4× MIC up to 55%. Because a large proportion of target isolates display MIC well below the breakpoint, microbiology laboratories should be encouraged to report actual MIC values rather than categorical susceptibility results, thereby enabling clinicians to make better-informed therapeutic decisions. Finally, patients requiring long-term therapy and/or those infected with pathogens exhibiting high MIC are at increased risk of developing resistance, irrespective of the administration schedule, and may therefore require alternative therapies taking into account the resistance profile of the offending organism.

**TABLE 3:** Proposed dosing recommendations for administration of meropenem using the OPAT-adapted regimen^*c*^

MIC of causative pathogen	PK/PD target			
	40% *f*T > MIC (non difficult-to-treat infection)	55% *f*T > MIC (difficult-to-treat infection)	55% *fT*fT > 4× MIC (difficult-to-treat infection, deep tissue)	100% *f*T > MIC (prevention of resistance)
MIC < 0.25 mg/L	q6h-6h-12h 20 min infusion	q6h-6h-12h 20 min infusion	q6h-6h-12h 20 min infusion	q6h-6h-12h 20 min infusion
MIC = 0.25 mg/L	q6h-6h-12h 20 min infusion	q6h-6h-12h 20 min infusion	q6h-6h-12h 20 min infusion	q6h-6h-12h 3 h infusion (portable pump)
MIC = 0.5 mg/L	q6h-6h-12h 20 min infusion	q6h-6h-12h 20 min infusion	q6h-6h-12h 20 min infusion	*q6h-6h-12h* *5 h infusion (portable pump)^a^*
MIC = 1 mg/L	q6h-6h-12h 20 min infusion	q6h-6h-12h 20 min infusion	q6h-6h-12h 1–3 h infusion (portable pump)	NA^*b*^
MIC = 2 mg/L	q6h-6h-12h 20 min infusion	q6h-6h-12h 20 min infusion	*q6h-6h-12h* *5 h infusion (portable pump)^a^*	NA
MIC = 4 mg/L	q6h-6h-12h 20 min infusion	q6h-6h-12h 1–3 h infusion (portable pump)	NA	NA
MIC = 8 mg/L	q6h-6h-12h 1-3 h infusion (portable pump)	*q6h-6h-12h* *5 h infusion (portable pump)*	NA	NA

^*a*^ 5 h infusion is NOT a standard practice, although meropenem stability is sufficient in these conditions.

^*b*^ Not applicable (this target cannot be reached).

^*c*^ The grey boxes indicate prolonged infusions (1h or 3h or 5h).

## MATERIALS AND METHODS

### Settings

This study (registered at EudraCT [number 2019-003900-10]) was conducted at the *Cliniques universitaires Saint-Luc* (*CuSL,* Brussels, Belgium), a 973-bed teaching hospital. Ethical approval was obtained from the *Comité d’Ethique hospitalo-facultaire* coordinating clinical studies at *CuSL* (unique Belgian registration number 2019/08OCT/431). Written consent was obtained from each patient or their legal representative, and patients were enrolled only once in the study.

### Study design, patients, and data collection

This single-center prospective, interventional academic study included non-critically ill patients hospitalized at the *CuSL*. The recruitment period spanned from November 18, 2019, to October 30, 2021. Inclusion criteria were as follows: (i) Adults ≥ 18 years (male or female), (ii) eGFR ≥ 50 mL/min (calculated using the CKD-EPI formula), (iii) requiring treatment with meropenem 2 g TID for a bacterial infection. Pregnant or breast-feeding women were excluded. Patients were included when their medical condition had stabilized. They were withdrawn from the study in case of: (i) modification of the treatment by the physician, (ii) lack of improvement under meropenem therapy, (iii) development of adverse effects related to the treatment, and (iv) consent withdrawal. Data collected included: (i) demographics (age, gender, weight, and body mass index), (ii) meropenem indication, (iii) microbiological data, and (iv) relevant biological parameters (C-reactive protein, serum creatinine, and eGFR).

### Antibiotic treatment and sample collection

Meropenem was administered for documented infections caused by Gram-negative bacteria with MIC ≤ 2 mg/L. Vancomycin was added when Gram-positive coverage was required. Two dosing regimens were used successively in each patient (Fig. 1): (i) standard regimen, that is, 2 g infused over 20 min in 50 mL of NaCl 0.9% TID (8 h interval between doses [q8-8-8 h]); (ii) OPAT-adapted regimen, that is, the same daily dose TID but administered during the day only (6 h interval between doses infused over 20 min [q6-6-12 h]). Spare sampling strategy was adopted because it was previously demonstrated to give PK parameter estimates consistent with those obtained with more intensive sampling (33). It consisted of collecting four blood samples for each dosing interval: for q8-8-8 h administration scheme, samples were collected 7 h 50 min after the previous dose and 1 h 15 min, 4 h 30 min, and 7 h 50 min post-dose; for q6-6-12 h administration scheme, sampling was performed 11 h 50min after the previous dose, 1 h 15 min, 4 h 30 min, 5 h 50 min after the morning dose, 5 h 50 min after the mid-day dose, and 1 h 15 min, 3 h 30 min, 11 h 50 min after the evening dose.

This OPAT-adapted scheme was adopted when the patient was clinically stable. Blood was collected via central or peripheral venous catheter in EDTA tubes, centrifuged to isolate plasma, which was stored at −80°C until analysis. HPLC analyses were performed within 30 days to avoid degradation; all samples from a given patient were analyzed simultaneously.

### Analytical method

#### Chemicals and reagents

Meropenem (powder for intravenous injection [Fresenius Kabi]) was obtained from the hospital pharmacy. The deuterated internal standard ([^2^H_6_]-meropenem) was purchased from Alsachim; HPLC-grade methanol and acetonitrile from J.T. Baker; formic acid and ammonium acetate from Merck KGaA. Ultrapure water was produced using either a MEDICA-R 7/15 system (Veolia Water Systems) or a Milli-Q Academic apparatus (Merck-Millipore).

#### Sample preparation

Serum was collected after blood centrifugation and frozen at −80°C. Samples were thawed at room temperature prior to processing. A volume of 500 µL of serum was transferred into Amicon 10 kDa ultrafiltration devices, which were then centrifuged at 14,000 rpm for 20 min. After centrifugation, the ultrafiltrate was collected into a Falcon tube and stored at −20°C until analysis. Before analysis, 30 µL of ultrafiltrate was mixed with 30 µL of internal standard ([^2^H_6_]-meropenem, stock solution at 10 mg/mL in methanol) and 40 µL of methanol.

#### Meropenem assay

Unbound meropenem plasma concentrations were measured on these ultrafiltrates, using a validated HPLC-MS/MS method, using [^2^H_6_]-meropenem as internal standard (10). The system consisted of a Quattro micro tandem mass spectrometer (Micromass) equipped with a Z-spray ion source, operated in electrospray positive ionization (ESI) mode and coupled to a Waters 2795 High Throughput HPLC System (Waters). Chromatographic separation was achieved using a Kinetex C18 column (100 Å, 50 × 3.0 mm, 2.6 µm particle size; Phenomenex) maintained at 30°C. The mobile phase consisted of 0.1% formic acid in water and 0.1% formic acid in methanol, delivered under isocratic conditions (30%/70%, vol/vol) at a flow rate of 0.15 mL/min. Quantification was performed using multiple reaction monitoring transitions (*m/z* 384 > 141.32 for meropenem and *m/z* 390.3 > 147.39 for [^2^H_6_]-meropenem). The cone voltage and the collision energy were set at 15 V and 15 eV, respectively, for both the analyte and the IS. The injection volume was 10 µL, and the total run time was 5 min. The autosampler temperature was set at 8°C. The validation of the method is presented in detail in our previous publication (10).

### Microorganisms and minimum inhibitory concentrations (MICs) determinations

MALDI-TOF MS (Microflex, Bruker Daltonics) was used for strain identification. Antimicrobial susceptibility testing was performed using a Phoenix automate (Becton Dickinson) for Enterobacterales and manual E-tests (bioMérieux) for non-fermenters and anaerobes. Interpretation of susceptibility was performed based on EUCAST and IDSA breakpoints (6, 15).

### Population pharmacokinetic analysis

Population pharmacokinetic modeling was performed using Monolix 2024R1 (Lixoft SAS, a Simulations Plus company) employing the stochastic approximation expectation-maximization (SAEM) algorithm. One- and two-compartment models with first-order elimination were evaluated as base models. Inter-individual variability (IIV) was described by an exponential model: $\theta_{j}=\theta_{p}\times exp(\eta_{j})$, where *θ_j_* is the parameter estimate for the *j*th patient, *θ*_p_ is the typical population value, and *η_j_*, a from a normally distributed random variable with mean zero and variance *ω*^2^. Residual variability was modeled using additive, proportional, or combined error models. Model selection was based on parameter precision, visual inspection of goodness-of-fit (GOF) plots, numerical assessment of objective function value (OFV), and corrected Bayesian information criteria (BICc).

After selecting the base model, covariate effects (body weight, height, gender, age, serum creatinine, and eGFR) were explored and retained if significantly improving the model fit (OFV decrease >3.84 (p< 0.05) for forward inclusion; OFV increase >10.83 (p< 0.001) for backward exclusion).

Model evaluation included goodness-of-fit (GOF) plots (observed vs population and individual predicted concentrations, NPDE vs population predictions and time), and visual predictive check (VPC) based on 500 simulations. The accuracy of the final model was assessed via a 1000-run bootstrap (34) using Monolix, comparing bootstrap medians and 95% confidence intervals.

### Monte Carlo simulation and probability of target attainment (PTA)

Monte Carlo simulations were performed using Simulx 2024R1 (Lixoft SAS, a Simulations Plus company) based on the final pharmacokinetic model. Steady-state (120-144h) unbound plasma concentration-time profiles were generated for the conventional regimen or the OPAT-adapted regimen.

For Gram-negative bacilli, the pharmacokinetic/pharmacodynamic (PK/PD) target was defined as the percentage of the 24-h period during which unbound drug concentrations exceeded the MIC at steady-state (*f*%T > MIC), with a threshold of 25–40% for bacteriostasis and 35–55% for a 1-log_10_ kill (6). In addition, a threshold at 100% *f*%T > MIC, suggested to lead to faster infection resolution in the critically ill patients, was also considered (16). Susceptibility breakpoints from EUCAST (2 mg/L for a standard dose of 1 g TID (not used here) and 8 mg/L for an increased dose of 2 g TID, that is, the dose administered to the patients included in this study) and from IDSA (1 mg/L for Enterobacterales and 2 mg/L for *P. aeruginosa,* with a recommended dose of 2 g TID except for non-complicated urinary tract infections [1 g TID]) (6, 15) were used as threshold values to evaluate dosing regimen adequacy.

### Statistical analysis

Normality of data distribution was assessed using the Shapiro–Wilk test. Parametric or non-parametric tests were applied based on distribution characteristics, with statistical significance set at p< 0.05. Data are presented as median [range], unless otherwise specified. GraphPad software version 10.5.0 (GraphPad Prism Software) was used.

## SUPPLEMENTAL MATERIAL

Supplemental material aac.00372-26-s0001.pdf Tables S1 to S3; Fig. S1 and S2.

ASM does not own the copyrights to Supplemental Material that may be linked to, or accessed through, an article. The authors have granted ASM a non-exclusive, world-wide license to publish the Supplemental Material files. Please contact the corresponding author directly for reuse.
