# Supplementary material for: A novel meropenem dosing strategy for Outpatient Parenteral Antimicrobial Therapy (OPAT): a pilot pharmacokinetic/pharmacodynamic study in non-critically ill patients
Source: Antimicrob Agents Chemother. 2026 Jun 12;70(7):e00372-26. doi: 10.1128/aac.00372-26 (PMC13321837; doi:10.1128/aac.00372-26)

A novel meropenem dosing strategy for Outpatient Parenteral Antimicrobial Therapy (OPAT): a pilot pharmacokinetic/pharmacodynamic study in non-critically ill patients.

Caroline Briquet, Perrin Ngougni Pokem, Xin Liu, Gert-Jan Wijnant, Olivier Cornu, Halil Yildiz, Alexia Verroken, Jason A. Roberts, Jean Cyr Yombi, Françoise Van Bambeke

Supplementary material

Table S1: Microbiological data (isolated bacterial species and meropenem MIC)

**Isolates (26)**	**Number of isolates with an MIC (mg/L) of**				
	**≤ 0.125**	**0.250**	**0.5**	**1**	**2**
Escherichia coli (6)	6				
Klebsiella pneumoniae (5)	5				
Enterobacter cloacae complex *(4)*	3	1			
Serratia marcescens (3)	2			1	
Proteus mirabilis (1)					1
Pseudomonas aeruginosa (2)					2
Serratia liquefaciens (1)					1
Acinetobacter baumannii (1)					1
Acinetobacter species (1)			1		
Bacteroides fragilis(1)	1				
Finegoldia magna (1)	1				
**Total, n (%)**	**18 (69.23)**	**1 (3.85)**	**1 (3.85)**	**1 (3.85)**	**5 (19.23)**

Table S2: Summary of time of exposure and lowest trough values for the different dosing regimens based on the population pharmacokinetic model

	***f*T>MIC**							
	**Conventional regimen**			**OPAT-adapted regimens**				
	**Time interval**	**q8-8-8h 20min infusion**	**q8-8-8h 3 h infusion**	**Time interval**	**q6-6-12h 20min infusion**	**q6-6-12h 1 h infusion**	**q6-6-12h 3 h infusion**	**q6-6-12h 5 h infusion**
	**MIC = 2 mg/L**							
**Individual doses**								
Dose 1	8h	>8 [2.6 - >8]	>8 [6.2 - >8]	6h	>6 [4.5 - >6]	>6 [4.9- >6]	>6 [5.8 - >6]	>6 [6 - >6]
Dose 2	8h	>8 [2.6 - >8]	>8 [6.2 - >8]	6h	>6 [4.6 - >6]	>6 [5.0 - >6]	>6 [6 - >6]	>6 [6 - >6]
Dose 3	8h	>8 [2.6 - >8]	>8 [6.2 - >8]	12h	10 [4.6 - >12]	10.5 [5.0- >12]	11.5 [6.2 - >12]	>12 [7.6 >12]
Daily	24h	>24 [14 - >24]	> 24 [12.6- >24]	24h	22 [14 - >24]	>22.5 [16 - >23]	>23.5 [18 - >24]	24 [20 - >24]
	**MIC = 8 mg/L**							
**Individual doses**								
Dose 1	8h	5.3 [4.6 - >8]	6.6 [4.0 - > 8]	6h	5.1 [2.6 - >6]	5.5 [2.9 - >6]	5.7 [4.0 - >6]	>6 [5.6 - >6]
Dose 2	8h	5.3 [4.6 - >8]	6.6 [4.0 - > 8]	6h	5.4 [2.6 - >6]	5.8 [3 - >6]	>6 [4.2 - >6]	>6 [5.6- >6]
Dose 3	8h	5.3 [4.6 - >8]	6.6 [4.0 - > 8]	12h	5.5 [2.6 - >12]	5.9 [3 - >12]	7 [4.2 - >12]	8 [5.6- >12]
Daily	24h	16 [14 - 24]	19.8 [12 - > 24]	24h	16 [9- >23]	17 [9 - >24]	>19 [12 - >24]	20 [17 - > 24]
	**Lowest Trough value (mg/L)**							
Dose 3	8h	3.2 [0.4-19]	5.3 [0.8-24]	12h	1.2 [0.1-11]	1.3 [0.1-11]	1.7 [0.2-14]	2.5 [0.3-16.5]

Table S3. PTA (in percentage) for different PK/PD targets and MIC comparing the simulated dosing regimens. Values > 90% are highlighted in green (this table shows the details of the data illustrated in Figure 3).

PK/PD Target	MIC (mg/L)	Dosing regimens					
		q8-8-8h 20min infusion	q8-8-8h 3h infusion	q6-6-12h 20min infusion	q6-6-12h 1 h infusion	q6-6-12h 3 h infusion	q6-6-12h 5h infusion
30% *f*T>MIC	2	100	100	100	100	100	100
	4	100	100	100	100	100	100
	8	97.9	100	97.9	98.9	100	100
	16	76.1	88.4	75.5	79.5	91.6	86.3
	32	29.1	38.6	27.7	31.8	41	38.4
35% *f*T>MIC	2	100	100	100	100	100	100
	4	99.3	100	99.3	100	100	100
	8	93.1	99.7	92.6	97	99.9	100
	16	66.1	82.1	66.2	71.6	83.8	84.6
	32	18.7	32.6	17.2	22.8	33.5	35.9
40% *f*T>MIC	2	99.9	100	99.9	100	100	100
	4	98.3	100	98.3	99.3	100	100
	8	86.1	99	85.5	91.6	99.4	99.9
	16	56.2	74.7	56.5	61.3	79.1	82.6
	32	12.4	24.1	12.5	14.8	26.7	33.3
45% *f*T>MIC	2	99.4	100	99.3	99.8	100	100
	4	95.8	100	95.5	98.1	100	100
	8	78.2	96.6	78.2	83.9	98	99.7
	16	43.6	65.8	44.2	52	71.9	81
	32	8.5	16.8	8.3	11.5	20.4	30.6
50% *f*T>MIC	2	98.3	100	98.3	99.3	100	100
	4	90.7	99.7	91.3	95	99.7	100
	8	69.5	93.6	71	76.6	93.9	99.5
	16	33	60.9	34.5	40.9	62.9	78.6
	32	4.6	14.6	5.9	7.6	15.2	26.9
55% *f*T>MIC	2	97.7	100	97.6	98.3	100	100
	4	86.4	99	86.4	91.2	99.1	100
	8	63.2	83.7	63.3	70	88.2	99.2
	16	27.8	49.6	27.9	33.2	54.1	75.9
	32	3.9	10.3	4.0	5.5	11.3	23.3
100% *f*T>MIC	0.125	99.7	100	95.6	96.8	98.8	99.7
	0.25	99.2	99.8	87.0	88.4	93.5	97.6
	0.5	95.0	99.0	72.5	75.0	82.3	90.5
	1	84.8	94.3	55.3	58.5	66.8	76.2
	2	67.4	81.3	31.9	35.2	44	57.4
	4	41.2	61	13.6	15.5	21	31.3
	8	16.3	30	3.8	4.2	6.9	10.9
	16	3.4	7.2	0.6	0.6	1.4	2.6
	32	0.3	0.7	0	0	0	0

Figure S1. Goodness of fit plots. (**A**) Observed versus population predictions (left) and observed versus individual predictions (right) plots for unbound plasma concentration; (**B**) Individual weighted residual (IWRES) versus time after dose (top-left) and individual predictions (bottom-left) and Normalized prediction errors (NPDE) versus time after dose (top-right) and population predictions (bottom-right). Closed circles: conventional dosing regimen; Open triangles: OPAT-adapted dosing regimen.

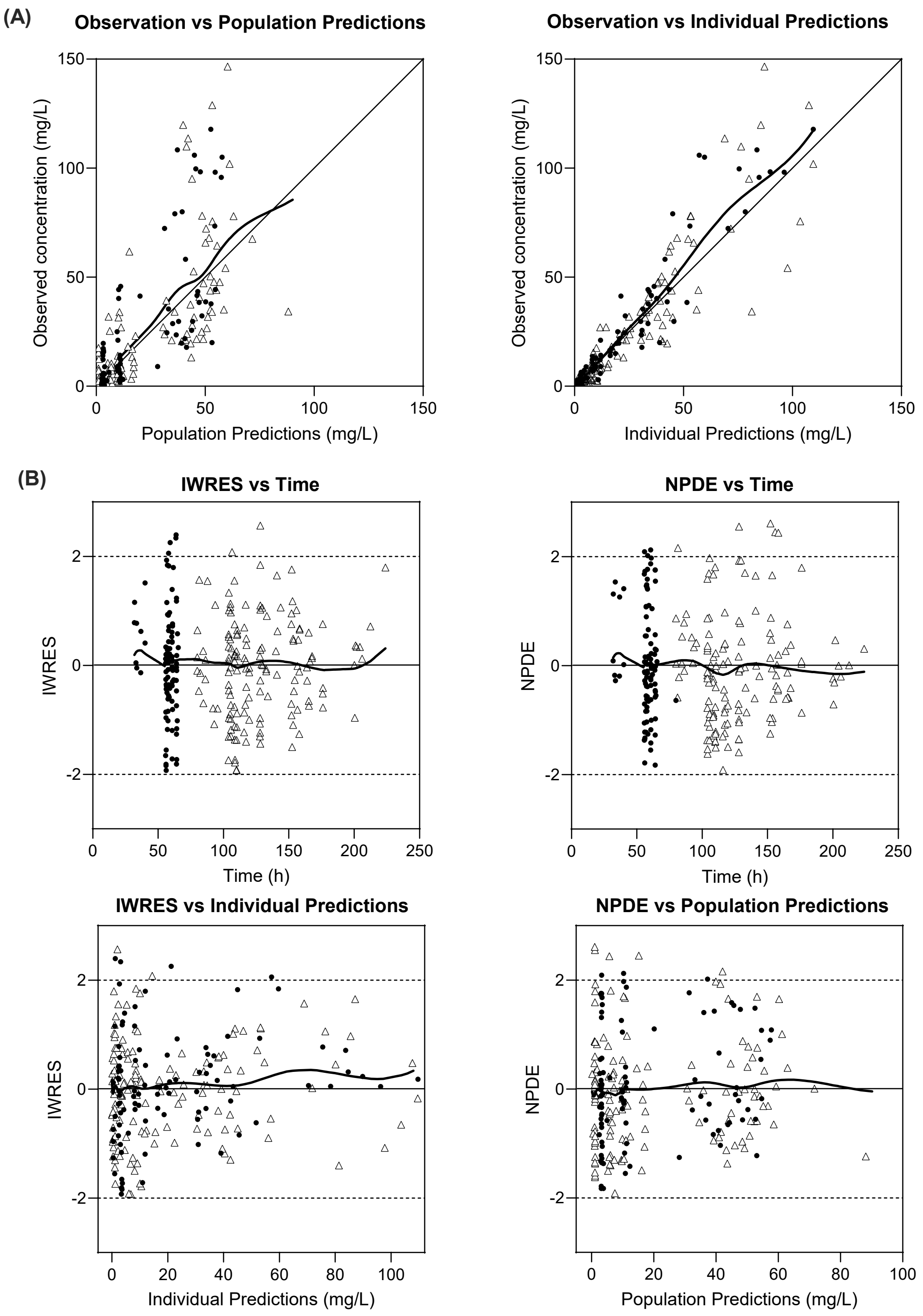

Figure S2. Visual predictive check (VPC) of the final model. Dots are observed concentrations, solid lines represent the 5th, 50th and 95th percentile of the observed values, and shaded area represents the 95% confidence intervals for the predicted percentiles. Closed circles: conventional dosing regimen; Open triangles: OPAT-adapted dosing regimen.

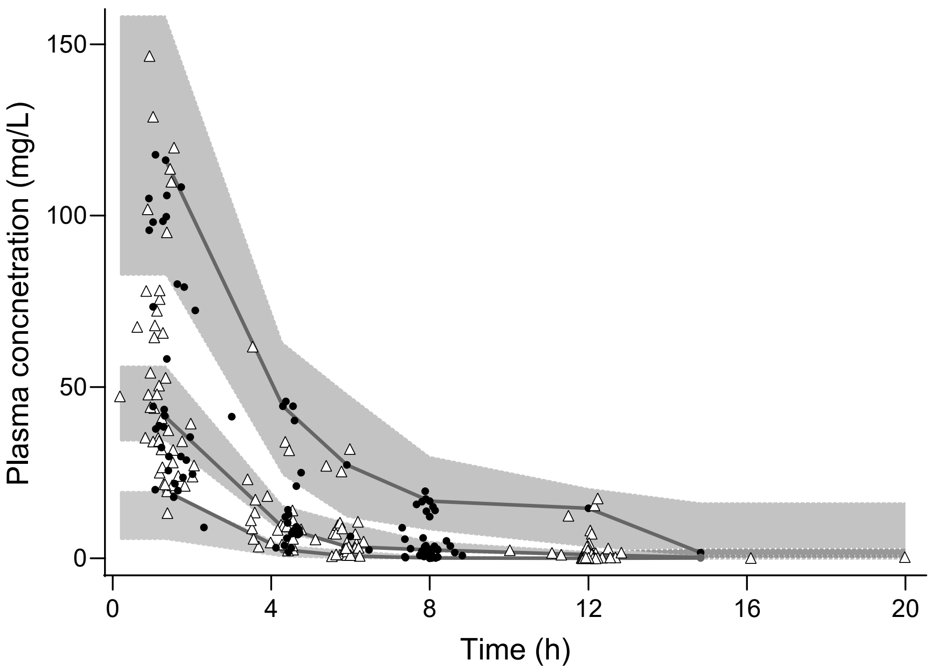

Supplement: Supplemental material — Tables S1 to S3; Fig. S1 and S2. [file aac.00372-26-s0001.pdf]
